# Developing an integrated model of community-based palliative care into the primary health care (PHC) for terminally ill cancer patients in Iran

**DOI:** 10.1186/s12904-021-00795-2

**Published:** 2021-06-28

**Authors:** Suzanne Hojjat-Assari, Maryam Rassouli, Maxwell Madani, Heshmatolah Heydari

**Affiliations:** 1 French Institute of Research and High Education (IFRES-INT), Paris, France; 2 Cancer Research Center, Shahid Beheshti University of Medical Sciences, Tehran, Iran; 3 Social Determinants of Health Research Center, Lorestan University of Medical Sciences, Khorramabad, Iran

**Keywords:** Palliative care, Community health care, Advanced cancer, Terminally ill, Home health care

## Abstract

**Background:**

Patients with cancer commonly experience pain and suffering at the end of life days. Community-based palliative care can improve the quality of life of terminally-ill cancer patients and provide them with a merciful death. The purpose of this study was to develop an integrated model of community-based palliative care into PHC for terminally ill cancer patients.

**Method:**

This study is a health system research (HSR) that was conducted in three phases from October 2016 to July 2020. In the first phase, dimensions of community-based palliative care were explored in patients with cancer using qualitative methods and conventional content analysis. In the second phase, a scoping review was carried out to complete the collected data from the qualitative phase of the study. Based on the collected data in the first and second phases of the study, a preliminary draft of community-based palliative care was developed for patients with cancer based on the framework of the World Health Organization. Finally, the developed model was validated using the Delphi technique in the third phase of the study.

**Results:**

Data analysis indicated that providing community-based care to patients with cancer is influenced by the context of care. According to the developed model, patients are identified as terminally ill, and then are referred to the local comprehensive health center in a reverse manner. After patients’ referral, they can receive appropriate healthcare until death by the home care team in relation to the comprehensive health center based on the framework of primary healthcare.

**Conclusions:**

This model was developed based on the current Iranian healthcare structure and the needs of terminally ill cancer patients. According to the model, healthcare is provided in a reverse manner from the subspecialty centers to patients’ homes in order to provide easy access to palliative care. It is suggested to use this model as a pilot at the regional level.

## Background

Prevalence of cancer is increasing in the world. According to the World Health Organization (WHO), its incidence has reached up to 18.1 million cases worldwide. According to this report, the mortality rate of cancer is approximately 9.6 million, which is considered as the second-highest cause of death in the world [1]. In Iran, the prevalence of cancer is also increasing and is considered as the second-highest cause of death in Iranians [2]. The WHO has introduced palliative care for promoting quality of life in terminally ill cancer patients [3]. Palliative care has a holistic view to patients and considers physical, psychological, social and spiritual dimensions [4]. This type of care not only helps patients maintain an active life until death, but also supports patients’ families over the trauma experienced due to the disease and/or death. It also results in a peaceful approach towards coping with death in families [3].

According to the WHO, palliative care and primary healthcare have common principles including continuity in care, social accountability, respecting patients’ values, and focusing on patients in the family. In the global health confederation, it was suggested that health systems should integrate home-based palliative care into primary healthcare in order to reach the goals of sustained development and universal health coverage (UHC) [5]. These services are provided by clinicians who know the patient and family [6]. Palliative care can be provided in a variety of places. One of the least expensive and most appropriate methods of palliative care is community- based and reliant on patient and family preference [7–11].

In a pilot study, palliative care was provided through primary healthcare to children with chronic conditions, which lead to increased access to care [12]. Home-based palliative care can result in an increase in quality of life, shorter length of hospitalization [13], less referrals to the emergency ward [14] and a higher chance of dying at home [15]. Access to home-based palliative care is one of the important components of sustained development and universal health coverage (UHC) [6].

Iran’s health system is based on differing levels of healthcare and referral, and healthcare is currently provided based on the primary healthcare (PHC) model. According to the structure of this system, comprehensive rural and urban health centers provide healthcare to specific regions [16]. Community-based palliative care as an innovative care approach has no place in this structure, and private and charity centers provide home-based care to the population under coverage [17].

Given patients’ preference to receive home care [18] and the successful structure of PHC in the country, it is necessary to include home care services in this structure as well. However, a proper model was not found that integrated home-based palliative service to terminally ill cancer patients based on the native and specific condition of the country. The purpose of this study was to develop an integrated model of community-based palliative care into the PHC for terminally ill cancer patients. If the model is designed, cancer patients can receive their care needs at the community level and in their place of residence.

## Methods

This study is a health system research (HSR) that was conducted in three phases from October 2016 to July 2020. It consists of three phases of qualitative, literature review and Delphi study.

### First phase of the study

This phase of the study was conducted qualitatively by using conventional content analysis in order to explore the context and dimensions of home-based palliative care for terminally ill cancer patients, the existing status of the health system and primary healthcare in Iran, and the integration of palliative care into the network system. Data were collected by purposefully sampling and interviewing 21 participants including oncologists, specialists in palliative medicine, general practitioners, nurses, psychologists, social workers, religious experts, family caregivers, cancer patients, and authorities of the network system and primary healthcare. Inclusion criterion for care providers was offering home care to advanced cancer patients for at least one year. For cancer patients, those with confirmed diagnosis of cancer, that received palliative care for at least three months in their homes were included. For faculty members, engagement in education and research about community-based palliative care, and for policy makers and managers, having at least one-year activity in the network system were regarded as inclusion criteria. Exclusion criteria were being unwilling and unable to participate in the study.

Data collection continued until the data saturated, so that no new code emerged from the interviews [19].

In addition to individual interviews, a focus group discussion was conducted with authorities and experts in home-based palliative care including oncologists, palliative care physicians, nurses and policy makers. The main questions guiding the interviews were different based on the knowledge, specialty and experience of the participants. The main questions were “tell me about home-based palliative care in Iran?”, “how was your experience about home-based palliative care?”, “tell me about the care of terminally ill cancer patients integrated into PHC?”, “what are the challenges and opportunities?”, “how can be facilitated?”. The investigator guided the interview using probing questions. In this study, data analysis and data collection was conducted simultaneously through Lundman and Graneheim [20]. After each interview, transcription was carried out verbatim and read several times. Then, preliminary codes were extracted. Preliminary codes that were related in terms of meaning were integrated and categorized based on similarities and differences. Finally, main categories emerged. For trustworthiness, credibility, transferability, dependability and confirmability were used according to Guba and Lincoln [21]. The investigator had long engagement with participants, which assists trust between them. Interpretation of codes were assured from participants’ perspectives. If codes were inconsistent with participants’ opinions, they were revised. Peer check was also used by two faculty members with expertise in qualitative research. In order to increase the credibility of data, we tried to enroll participants in a way that maximum variation in knowledge, working experience, position, specialty, age, and gender is fulfilled.

### Second phase of the study

In the second phase of the study, the research team conducted a comprehensive literature review using a scoping review approach, used to enrich the data previously collected in the first phase of the study. This section of the study aims to examine healthcare services provided to terminally ill cancer patients. Science databases like Scopus, Medline/PubMed, and Google scholar were searched without a time limit, using keywords such as ‘end of life’, ‘palliative care’, ‘cancer patient’, ‘home care’, and ‘model’ limited to English language.

**Fig. 1:**
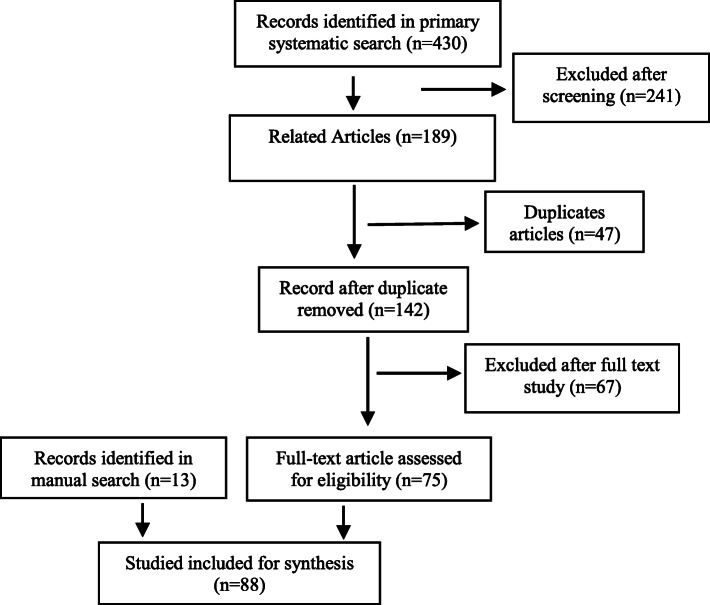
PRISMA diagram for reporting search results

Persian articles were found on Google scholar, Magiran, Sid, and Iran doc by searching the aforementioned keywords on the database search engine in Farsi. After retrieving and selecting the articles, data was extracted and examined using qualitative synthesis [22]. The references of all selected articles were also reviewed, in order to improve the quality of the data that are appropriate according the aims of the study. Moreover, grey literature in the form of reports from the WHO website was retrieved. Searching through the google search engine led to the identification and selection of several additional works of grey literature (conference summaries, unpublished research projects, theses). Wherever possible, we used the PRISMA criteria to report items (Fig. 1).

The selected articles were read, and phrases mentioned in the articles were labeled using codes. The codes emerged during the literature review were integrated with the codes extracted from the qualitative phase of the study and categorized based on their differences and similarities.

### Developing draft of integrated model of community-based palliative care

The purpose of this phase was to develop an integrated model of community-based palliative care into the PHC for terminally-ill cancer patients. By the end of the second phase, a draft of the healthcare model for terminally-ill cancer patients was developed using concepts extracted from the qualitative phase and the scoping review based on the integrated model of palliative care into the PHC introduced by the WHO. Then, credibility of its main dimensions were determined from the perspective of authorities using a questionnaire with the answer choices: completely disagree (1), disagree (2), agree (3), completely agree (4).

### Third phase of the study

#### Credibility of the developed model

The validity of the developed model in terms of feasibility was assessed using the Delphi technique. The participants who were recruited in the first phase of the study also participated in the third phase of study. Electronic files or hardcopies of the questionnaire were provided to 21 key informants who were asked to fill the model during a two-week period. From the questionnaires sent, 18 questionnaires were filled and returned to the investigator. In the third phase of the study, the collected data was analyzed using SPSS (2019, Version 26). Mean and standard deviation of each question were determined. The research team assessed each participants’ opinions and suggestions mentioned in the questionnaire. In this study, scores with a mean of 3 or above meant that the participant considered the proposed model as credible. Scores below 3 illustrate that those criteria needed to be excluded or revised. The criteria that the expert panel suggested to revise were ultimately changed.

#### Ethical considerations

This study was approved at the Ethics Committee at Lorestan University of medical sciences “LUMS.REC.1394.57” and “IR.LUMS.REC.1398.217”. The investigators considered the ethical principles of obtaining written consent from participants, the confidentiality of data, and the anonymity of participants in all phases of the study.

## Results

### Findings of the qualitative phase of the study

Qualitative data in this phase was collected through 21 face-to-face interviews with 12 men and 9 women (Table 1) and a focus group discussion including six men and two women (Table 2).

**Table 1:** Participants in the individual interviews

Number	Age years	Highest level of education	Working experience years	Position
**1**	62	Specialist	30	Active in policy making and home care
**2**	48	Specialist	25	Active in policy making and home care
**3**	38	Nurse	16	In charge of coordination of home-based palliative care
**4**	52	PhD in Nursing	26	Lecturer of public health nursing
**5**	47	Nurse	19	In charge of palliative care ward
**6**	46	Theological	6	religious expert
**7**	45	Nurse	16	Caregiver of home-based palliative care
**8**	43	Medical doctor	16	Palliative care physician
**9**	43	Social worker	11	Social worker
**10**	32	Staff	-	Caregiver
**11**	25	Diploma	3	Assistant
**12**	34	General practitioner	4	Palliative care physician
**13**	53	Nursing assistant	8	Nursing assistant in the palliative care ward
**14**	33	Psychology	3	Psychologist in home-based palliative care
**15**	29	Psychology	5	Psychologist in home-based palliative care
**16**	40	Bachelor in nursing	17	Home care nurse
**17**	38	Bachelor in nursing	10	Home care nurse & in charge of home care institute
**18**	38	PhD in health policy	6	Faculty member of health and health promotion
**19**	42	PhD in epidemiology	17	Staff of the Health Deputy
**20**	37	Pharmacist	5	In charge of drugs used in the network system
**21**	54	Staff	24	In charge of narcotics

**Table 2:** Participants in the focus group discussion

Number	Age Years	Leve of education	Working experience Years	Position
**1**	62	Specialist	32	Active in home care
**2**	51	Specialist	18	Active in home care
**3**	48	Social medicine specialist	19	Representative of the Ministry of Health
**4**	47	Doctor	23	Agent of Iranian Health Insurance
**5**	54	Master in nursing	30	Manager of home care institute
**6**	42	Bachelor in Nursing	16	Home care nurse
**7**	44	Social medicine Specialist	8	Active in home care policy making
**8**	42	PhD in nursing	15	Active in home-based palliative medicine

Qualitative data analysis was viewed through three main categories including the structure of the health system in terms of opportunity, outcomes and requirements. Each category had a few subcategories (Table 3).

**Table 3:** Categories and subcategories emerged in the qualitative phase of the study

Category	Subcategory	Codes extracted from the interviews	Quotations
Structure of the health system as an opportunity	Using the PHC principles in the healthcare system	Access to the comprehensive health centers for all; healthcare providers have access to the records of patients with cancer. In the comprehensive health centers, healthcare is provided based on the PHC principles, variety in health staff in the comprehensive health centers, families have close relationship with the comprehensive health centers.	“… one of the programs that is very important and can be employed in the PHC framework is care of patients with cancer … [18]”.
	Exchange of information electronically	Existence of an integrated electronic health system to record people’s information into the health system; the electronic exchange of information in all sections of the Ministry of Health; access to the information of all families through an integrated electronic health system	“… the ideal state is one in which the integrated electronic health system is linked to patients’ information in hospitals. Consequently, referral and patient care programs are performed easily … [18].
	Establishment of the referral system	There is a referral system in rural areas and small towns. The referral system is a priority for the Ministry of Health. The referral system is carried out as a pilot in several provinces. The referral system can be used for cancer patients as a reverse referral.	“… In the care of patients with tuberculosis and leprosy … are identified in higher level … from a subspecialist and specialist is referred reversely … [18]”.
	Establishment of family physician	Establishment of a family physician in rural areas and small towns, coverage of a specific population by family physician, cancer patients are also under the covered population. The plan of family physician includes performing in several provinces as a pilot.	“… family physicians cover the population. Any individual who needs palliative care is under the coverage of one of these physicians so … family physician should be included in the palliative care … [18].”
Requisites	Home care team in palliative care at the PHC level	Necessity of defining a specific population for a home care team, necessity of defining a home care team in the PHC structure; necessity of communication between a healthcare team and comprehensive health centers, delivery facilities that exist in the PHC can be used as a model. A home care team can support cancer patients effectively.	“… in this case, I remember delivery facilities and minor surgery centers, … accessible for 20,000 population … in 5–6 health centers … in a health center, we establish delivery facilities … [18]”.
	Narcotic management	Necessity of pain management in patients with cancer; necessity of appropriate structure to regulate access to narcotics; necessity of a structure to monitor usage of narcotics in patients	“… we can provide three groups of narcotics including morphine, methadone, and Pethidine to patients based on the monthly prescription … [21]”.
	Equipment management	Terminally ill cancer patients need various equipment. Necessity of defining appropriate structure for equipment management; necessity of easy access to the equipment for patients; patients can borrow equipment.	“… portable suction … before transferring the patient to home, bed and equipment should be provided … patients can borrow these [17].”
	Social coverage	Necessity of home-based palliative care covered by insurance; defining tariff of home-based palliative services; one of the barriers of access is financial issues.	“… all services should be free or with little franchise … for example, patients with diabetes or hypertension. Indeed, less is paid by patient … [19]”.
	Legal issues	Necessity of considering legal issues; necessity of defining moral guidelines for care of terminally ill patients; necessity of developing a deal between families and healthcare centers	“… the first thing to commit both the center and patient is an agreement … [19]”.
Outcomes	Facilitating access	Easy access to healthcare; facilitating access based on what international organizations emphasize.	“… when a service is placed in the PHC structure, it is expected to be free. Indeed, financial access is provided …” [18].
	Good death	Providing proper conditions for peaceful death; using psychological consultation for facilitating death; dying at home for patient and family is easier.	“… with comfort provided to the family using a psychologist, using various people can improve quality of death …” [18].

### Findings of the scoping review

In the scoping review, 430 articles in English were retrieved from various databases. After reading the titles and abstracts of articles, 75 articles were used based on their relevancy to the objective of the study. In the manual search, 31 articles and manuscripts were retrieved. Then, the full texts of 13 articles and manuscripts were appraised. Finally, in both the manual and systematic search, 88 articles and manuscripts were appraised (Fig. 1).

Data analysis in the scoping review section was carried out with three main categories (inter-sectional corporation, teamwork services, and providing optimum care, good death) and three subcategories (structure of home care team, family conferences, and criteria of patients’ assessment (Table 4).

**Table 4:** Categories and interpretation of data in the literature review section

Category	Subcategory	Codes extracted from the literature	Texts from references
Inter-sectional corporation		Necessity of cooperation in all levels of prevention in the Ministry of Health; necessity of cooperation of the Ministry of Health with other governmental and non-governmental organizations; necessity of providing care in private and public outpatient clinics	Inter-sectional corporation such as public hospitals, nongovernmental organizations, and charities [23]. Services to patients can be provided in clinics and consultation centers [24].
Providing optimum care to patients	Structure of home care team	Cancer patients should receive comprehensive care from a home care team; palliative care should be provided by various caregivers with different specialties to patients; home care team composed of various specialists.	Palliative care team can consist of oncologist, palliative care physicians, nurses at different levels of care, social workers, religious experts and other healthcare providers [25].
	Family conference	Providing information to the family about their role in care team, providing information to the family in different stages of the disease, explaining the benefits of transferring the patient to home care to the family, making decision about the patient with family	In order to provide home care, the patient and family should be prepare to confront the problem in different stages of the disease [26, 27].
	Criteria of patient assessment	Using standard tools for identifying needs and decision-making for the patient’s care	Palliative performance scale (PPS) can be used as a good index of predicting the survival and health status of the patient [28, 29].
Good death		Paying attention to the preferences of patients and families can influence a good death. Healthcare team assistance can facilitate tolerance of patient’s death for family.	Determinants of death at home for terminally ill cancer patients include patient preference, family support; support from health systems after death of these patients at home, desire to using home care; policies of health system regarding palliative services; cultural and ethnical differences [30–36]. After the death of a patient with cancer, bereavement is carried out for 13 months for the patient’s family [37]

At the end of the second phase, a preliminary draft of community-based palliative care was made using the collected data from the qualitative phase and literature review. Then its credibility from the perspectives of authorities, the main domains were developed as questionnaire from completely disagree (1), disagree (2), agree (3) and completely agree (4). This questionnaire consists of 11 domains and 69 questions on a 4-point Likert scale. The domains include: 1. Process of admission and transfer (11 questions) 2. Inter-sectoral and interprofessional cooperation (8 questions) 3. Human resources (8 questions) 4. Narcotic management (4 questions) 5. Equipment management (5 questions) 6. Social support (3 questions) 7. Legal issues (12 questions) 8. Family conference (4 questions) 9. Home care team (5 questions) 10. Continuous assessment of the patient (3 questions) 11. Death management (6 questions). Score of each question ranges from 1 to 4. The questions that received a mean score of 3–4 remained, and those with a mean score of less than 3 were excluded or replaced with new questions. After obtaining supplemental information from the participants, the suggestions were accepted or rejected.

### Findings of the third stage of study

The preliminary draft of home-based palliative care was developed as a questionnaire. It was then provided to 21 authorities. Eighteen questionnaires were completed and returned. The results showed that 10 men and 8 women with the mean age of 48.83 ± 8.14 years and 23.44 ± 8.08 years of work experience took part in the study. After data analysis, the subjects obtained scores from 3.35 ± .67 to 3.96 ± .73 for 61 out of the 69 questions. Of these, the score of 8 questions were between 2.11 ± .51 and 2.22 ± .73, which were then excluded or revised. Of these 8 questions, 2 questions were in the inter-sectoral collaboration, 1 question was in the management of narcotic drugs, 4 questions were about the home care team, and 1 question about the continuous assessment of patients. The research team also interpreted all suggestions and opinions and revised or excluded questions accordingly.

In each province of Iran, universities of medical sciences are responsible for health and treatment. One of the most important deputies in each university is the Health Deputy, who provides services through health centers in various cities. Health centers in each city provide services to urban populations through comprehensive health centers. Populations under coverage of each comprehensive health center is between 25,000 and 50,000 people, which is distributed under 3–5 comprehensive health bases. Staff in each comprehensive health center includes physicians, nurses, specialist staff (psychology, nutrition and health), an internal manager and guards [38]. Comprehensive health centers are responsible for various tasks. The model of home-based palliative care for terminally ill cancer patients requires referral services from health bases including medical services, medications, nursing, nutrition, physical activity, mental healthcare, follow up of noncontagious diseases and active participation of the community. Each comprehensive health center covers 3–5 health bases. Each base covers 8000–12,000 people based on the density of the populations Health bases provides various services including health promotion, health education, nutrition, home visit, screening, collaboration with others and referral using healthcare providers, nurses, and midwives.

### Model

According to the findings of this study, an integrated model of community-based palliative care with PHC was designed. All parts of the model were adjusted to the upstream rules of Iranian health system on providing home-based palliative care to cancer patients. According to the World Health Organization’s requisites, patients’ end of life needs has been taken into consideration in this model, including management of physical, psychological, social and spiritual needs.

It is expected that cancer patients to be able in addition to being supported by the third and second levels of referral system also receive the palliative care services in the first level. The focus of the current study was on the reverse referral of patients from subspecialty centers in the third level to comprehensive health centers in the first level of the referral system.

**Fig. 2:**
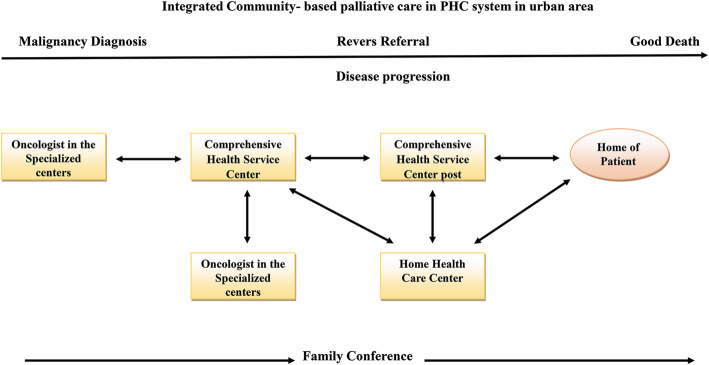
Integrated Community-based palliative care in PHC system in urban area

The starting point of this model is the entrance of patients into the palliative phase based on an oncologist’s diagnosis. A family conference is held for the family by attending the oncologist. Patient and family consent is obtained in order to continue treatment at home. Then the patient’s record is referred electronically or physically to local comprehensive health centers (Fig. 2).

- A: Tasks of comprehensive health centers: forming a palliative and supportive record; performing first palliative, supportive and medical consultation for recording medical information and determining patient’s performance based on international standards such as Karnofsky Performance Scale; assessment of physical, psychological, social and spiritual needs; determining the treatment path; family conference for announcing treatment plan. After these consultations, subsequent consultations are performed on an outpatient basis. In this stage, the patient is referred to a health base.
- B: Tasks of health base: a health base maintains a close relationship with patients; management of referrals for home care; assessment of social needs, social work services, patient and family education. If the comprehensive health center recommends, medical, nursing, and other consultations can be planned in the health base. Palliative services continue through coordination with comprehensive health centers until patient’s performance allows.
- C: Home care centers: home care centers can be nursing centers or specialist palliative supportive centers. In terms of the service level, these centers meet patients’ needs at home.
- D: General hospitals: in the diagnosis of emergency situations or the patient’s need for advanced care including physical symptom management, pain management, or surgery, then the home care team refers the patient to the comprehensive health center. The physician of this center can refer the patient to the local or regional general hospital or public clinic. After meeting the therapeutic needs of the patient and stabilizing their physical status, he or she is referred to the comprehensive health center and/or home.
- E. Information management: in each stage of these services, information is recorded in the palliative and supportive file of patient. This information is accessible to all levels of patient referral. Coordination between home care centers, comprehensive health centers, and comprehensive health bases is the key to success in providing supportive palliative services.

## Discussion

In this study, a model of home care to terminally ill cancer patients integrated into the primary health care was developed. Process of care was considered in a reverse manner from the tertiary level of prevention to patients’ homes. Consistent with the finding of this study, in a model of community-based palliative care in Busan Korea, health centers were considered as the central hubs of providing care [37]. In Western North Carolina, a Season’s Community-Based Palliative care model was designed in which an inter-disciplinary team provided care to patients in both outpatient and inpatient settings, including psychosocial/spiritual care, advanced care planning, symptom management, and patient/family education [2]. Also, similar to the model designed in this study, a palliative care model was designed in European countries to provide services to patients by specialized teams at home and clinics [39].

The developed model is based on the definition of the PHC according to the WHO, in which palliative care should be provided by health centers so that it is accessible and cost-effective to all people [40]. And also results of a study showed Community-based palliative care was cost-benefit for health systems [41]. Increased people’s access to health services is consistent with the goals of sustained development and universal health coverage (UHC) [5].

The developed model is performed in the reverse referral path so that sources of referral are central and specialist units, and destinations are comprehensive health centers and general hospitals. In Sarvak’s model, patients are referred from specialist centers to the coordinator nurse of home care teams. Then, they are referred to the local health centers with a care program [42]. In this model, patients flow between the primary, secondary, and tertiary levels of the health system. The focus of the model is to maintain patients at home and for them to receive healthcare from the local health centers. WHO has emphasized integration of palliative care into the health system, particularly in the community in which palliative care is provided by the network of care teams [5]. Consistent with this study, Nabudere’s model, palliative care is provided at various levels of the health system [43].

In the developed model, employed staff in comprehensive health centers are considered to manage human resources. Comprehensive health centers in Iran, which perform healthcare based on the PHC, have a combination of human resources for providing palliative care. Health bases also have the potential of health connectors interacting with people. In line with the findings of this study, the model developed in Korea is comprised of existing human resources that were used by modifying their work instead of increasing the size of their work force [37]. It seems that the plan can be managed through minor changes in the structure of human resources and the revision of work done by healthcare providers. One of the deficits in comprehensive health centers is that no nurse is available in these fields [44]. While in the Four Season’s Community-Based Palliative care model nurses act in the team as care provider, manager and coordinator [39]. Then it is a necessity that nurses in the Iranian health system be employed for providing community-based services.

In the designed model, access to narcotic drugs for controlling patients’ pain was defined in the structure of comprehensive health centers. In line with the findings of this study, in Sarvak’s model, morphine is prescribed for patients with cancer for 2–4 weeks. For patients who live in the suburb, drugs are prescribed by an oncologist for 3 months [42]. In Morrison’s model, oral morphine is used by some health staff including physician and nurse in community palliative care [45]. In Iran, management of narcotics is based on the guideline of narcotics consumption and distribution with responsibility of a university of medical sciences. The Health Deputy can provide context for the delivery of narcotics to patients in comprehensive health centers [46].

Another important point in the designed model is the renting of required equipment for patients.

Proper management of stock including bed, oxygen and suction can be achieved by lending them to patients [42]. After the patient’s death, the equipment was provided to others with consideration of safety and infection control.

In the developed model, providing care is defined as teamwork at different levels of the health system, from the primary level of prevention in the community to the tertiary prevention level in specialist hospitals. Moreover, nongovernmental organization and charities have a role in the process of providing care to patients. In line with this study, the findings of another study indicated that providing care to these patients requires proper coordination and cooperation among all fields of healthcare sectors including the hospital, the physician responsible for primary health care, and patients’ homes [47]. Another important point that affects quality of care is the involvement of a patients’ family in planning care. Over this process, a patient’s family should be trained. In this process, the patient and family should communicate with the coordinating physician continuously [48]. The patient’s family can support the patient to prevent the progression of the disease and to become prepared for death [49, 50].

Since the mortality rate in cancer patients is 55,785 per year in Iran, and Iran’s population is 82,011,737 in 2018 [51], death due to cancer can be viewed as 68 deaths per 100,000 people in the population. It seems that a home care team can cover patients under coverage of a comprehensive health center with 100,000 people. In line with the findings of this study, in the European model of Palliative Care, one home care team is considered for 100,000 inhabitants [39], and in the Korean model, 42,000 individuals are covered with one home care team [37]. The difference in population coverage by a home care team in two countries could be explained by differences in incidence and mortality rate of cancer [52]. It seems that in the developed model, both home care teams can have a social worker and a religious expert. Authorities of the comprehensive health centers are responsible for monitoring and managing these teams [37]. During providing palliative care to patients and families, the course of the disease is progressive. Over this progression, the patient’s autonomy decreases, and their care needs increase. An index of palliative performance scale [53, 54] or other standard criteria can be used for assessment and determination of patient’s needs.

Another important point in the designed model is offering patients a good death. This area is consistent with the definition of palliative care according to the WHO [55]. In line with findings of this study, Fan et al. points out that a healthcare team should be informed about the patient’s remaining duration of life, prognosis, expected signs, outcome and complications of resuscitation and the patient’s preferences [56]. In the developed model, staff of comprehensive health centers and home care teams are involved with patients and their families from the early stages of the disease. Healthcare providers can provide context to patients regarding a good death and can help families tolerate the loss of a close relative. Spiritual care can also assist these patients and should be included in care programs.

One of the limitations of this study is that palliative care and home care in Iran have emerged in the healthcare system over recent decades; therefore, the lack of authorities and infrastructure are the most important limitations of this study.

## Conclusion

The model of community-based palliative care to terminally ill cancer patients integrated to primary health care was developed based on Iran’s healthcare system and needs. The process of care was considered in a reverse manner from the tertiary level of prevention in the specialist hospitals to primary level of prevention in the community and patients’ home.

Patients have easy access to the end of life care based on the principles of PHC. During the process of care, family can be prepared for the patient’s death, which will make the grieving process more tolerable. It is suggested that this model be used as a pilot in the real world, especially the field of primary health care, in order to determine its strengths and weaknesses. After various tests and filters in the real field and recognizing and fixing its weakness, it can be used as model of community-based palliative care to terminally ill cancer patients in Iran’s healthcare system.

## Data Availability

This study conducted by qualitative method, audience and text file are available from the corresponding author if needed.

## Abbreviations

PHC: Primary Health Care
HSR: Health System Research (HSR)
WHO: World Health Organization
UHC: Universal Health Coverage
